# Computed tomographic features of adenoid cystic carcinoma in the palate

**DOI:** 10.1186/s40644-019-0190-z

**Published:** 2019-01-31

**Authors:** Wu-tong Ju, Tong-chao Zhao, Ying Liu, Yi-ran Tan, Min-jun Dong, Qi Sun, Li-zhen Wang, Jiang Li, Lai-ping Zhong

**Affiliations:** 1 Department of Oral and Maxillofacial-Head and Neck Oncology, Ninth Peoples Hospital, College of Stomatology Shanghai Jiao Tong University School of Medicine, No. 639 Zhizaoju Road, Shanghai, 200011 China; 2 Department of Oral Radiology, Ninth Peoples Hospital Shanghai Jiao Tong University School of Medicine, No. 639 Zhizaoju Road, Shanghai, 200011 China; 3 Department of Oral Pathology, Ninth Peoples Hospital, College of Stomatology Shanghai Jiao Tong University School of Medicine, National Clinical Research Center for Oral Diseases Key Laboratory of Stomatology, Shanghai, China

**Keywords:** Palate, Adenoid cystic carcinoma, Salivary gland tumors, Prediction model, Computed tomographic features

## Abstract

**Background:**

To evaluate the computed tomographic features and create a prediction model for clinical diagnosis of adenoid cystic carcinoma (ACC) in the palate with intact mucosa.

**Methods:**

From March 2016 to May 2018, 102 patients with palatal tumors and intact mucosa, including 28 patients with a pathological diagnosis of ACC after surgery, were enrolled in this study. The patients’ clinical symptoms, computed tomographic features and pathological diagnoses were recorded and analyzed. Independent predictors of ACC were determined by using univariate analysis and multivariate logistic regression, and the discrimination and calibration of the prediction model was evaluated, and internal validation was performed.

**Results:**

Univariate analysis of patients showed that ACC patients were more likely than non-ACC patients to be older (*P* = 0.019); to have palatine bone destruction (P<0.001) and greater palatine foramen (GPF) enlargement (P<0.001); to have involvement of the pterygopalatine fossa (P<0.001), foramen rotundum (P<0.001), nasal cavity (P<0.001) and maxillary bone (P<0.001); and to have numbness (*P* = 0.007) and pain (P<0.001). Multivariate logistic analysis showed that age and GPF enlargement were independent predictors of ACC in palatal tumors. The diagnostic prediction model showed good discrimination and calibration, as evaluated by the area under the receiver operating characteristic curve (0.98) and the Hosmer-Lemeshow goodness-of-fit test (*P* = 0.927).

**Conclusions:**

The palate ACC prediction model based on age and GPF enlargement shows excellent discrimination with no evidence of poor calibration. Older patients with palatal tumors and intact mucosa should be considered for ACC when they have GPF enlargement.

**Electronic supplementary material:**

The online version of this article (10.1186/s40644-019-0190-z) contains supplementary material, which is available to authorized users.

## Background

Adenoid cystic carcinoma (ACC), a common malignant tumor originating from the salivary glands, is characterized by slow growth and perineural extension [1]. The overall 10-year survival outcome for ACC is approximately 50%, and locoregional and distant recurrence is common after a disease-free interval [2]. Surgical resection is the mainstay of treatment for patients with primary ACC [3]; however, a variety of studies have shown that definitive radiation might result in reliable control of this disease [4–6].

More than half of ACC cases arise from the minor salivary glands, of which the palate is the most common location [7–9]. In the palate, the three most common types of salivary gland tumors are pleomorphic adenoma (PA), mucoepidermoid carcinoma (MEC) and ACC [10, 11]. Patients with these types of palatal tumors present with a mass with similar manifestations, such as swelling, a lack of tenderness and fluctuance, a hard consistency or immobility, and occasional pain or numbness [12–15]. When patients with palatal tumors have intact mucosa, making accurate differential diagnoses among these tumors on the basis of physical examinations is sometimes difficult. Moreover, other types of tumors can occur in the palate, such as myoepithelioma, carcinoma ex-pleomorphic adenoma, acinic cell carcinoma and lymphoepithelial carcinoma (LEC), thus making clinical diagnosis even more difficult.

Accurate clinical diagnosis of the palatal mass is very important in developing treatment plans. Biopsies are easy to perform in the oral cavity. However, when the mass is located in the palate with intact mucosa, incision biopsy is not recommended, and fine-needle aspiration might be critical for accurate identification of ACC in salivary glands [16]. Positron emission tomography-computed tomography has been reported to be useful for demonstrating the stage of ACC, but it is costly [17]. Diffusion-weighted magnetic resonance and diffusion tensor imaging are useful for the characterization of and differentiation between benign and malignant salivary gland tumors but have not been specifically reported in the diagnosis of ACC [18, 19]. Computed tomography (CT) and magnetic resonance imaging (MRI) are considered reliable and convenient methods for diagnostic and prognostic prediction [20–25]. They are also recommended to detect perineural spread (PNS), a critical feature of ACC [26–29]. However, no previous systematic diagnostic study of ACC based on CT or MRI features has been reported. The objective of this retrospective study was to analyze the CT features of ACC in the palate with intact mucosa, and to develop a prediction model for clinical diagnosis of ACC on the basis of physical and imaging examination results.

## Methods

### Patients

**Fig. 1:**
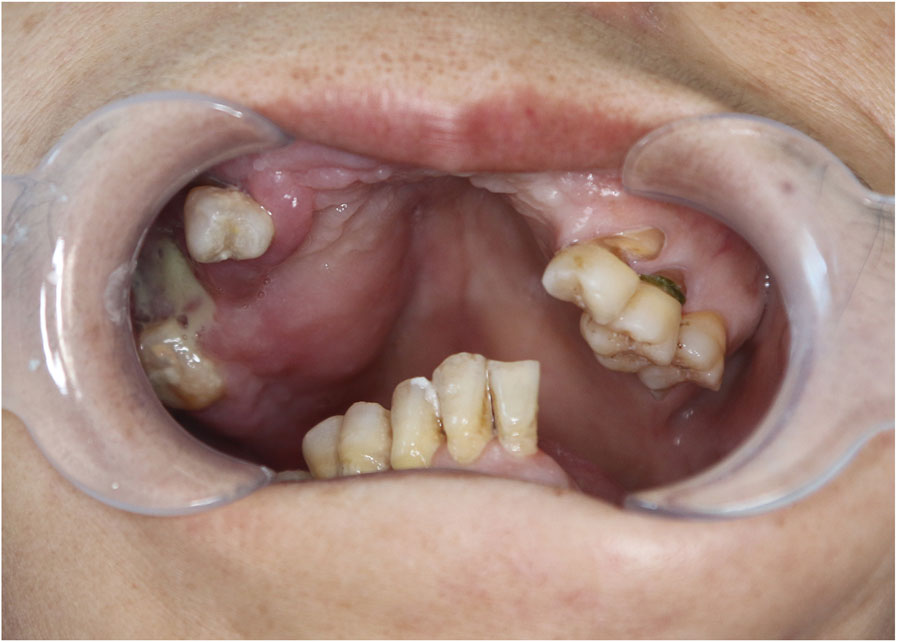
Intraoral photograph of a palatal mass. A patient with adenoid cystic carcinoma (hemispherical region of swelling) in the palate with intact mucosa

From April 2016 to May 2018, 102 patients with palatal tumors and intact mucosa, who presented with a similar chief complaint (Fig. 1), were enrolled in this study. All patients were treated at the Department of Oral and Maxillofacial-Head and Neck Oncology, Ninth People’s Hospital, Shanghai Jiao Tong University School of Medicine. This study was approved by the hospital ethics committee. Informed consent forms, which had been approved by the ethics committee and the institutional review board of the hospital, were obtained from all patients.

The patients’ general condition, clinical manifestations and symptoms, CT features and pathological diagnoses were reviewed and analyzed. All patients had received clinical examination, a CT scan and surgical treatment. Patients with squamous cell carcinoma in the palate were excluded because this cancer usually presents palatal or gingival ulceration, tooth looseness, numbness and headache, rather than palatal swelling with intact mucosa. Patients who had recurrent tumors or tumors with incomplete mucosa or ulceration, or who did not undergo contrast-enhanced CT or surgery, were not included in this study (see Additional file 1). Clinical manifestations and symptoms were recorded at the time of hospitalization, and the CT images were evaluated by two experienced radiologists. Clinicians and radiologists were blinded to the results of the pathological diagnosis, and consensus readings were performed in the case of interpretation discrepancies. The pathological diagnoses of excised tumors were confirmed by two experienced oral pathologists.

### CT features

**Fig. 2:**
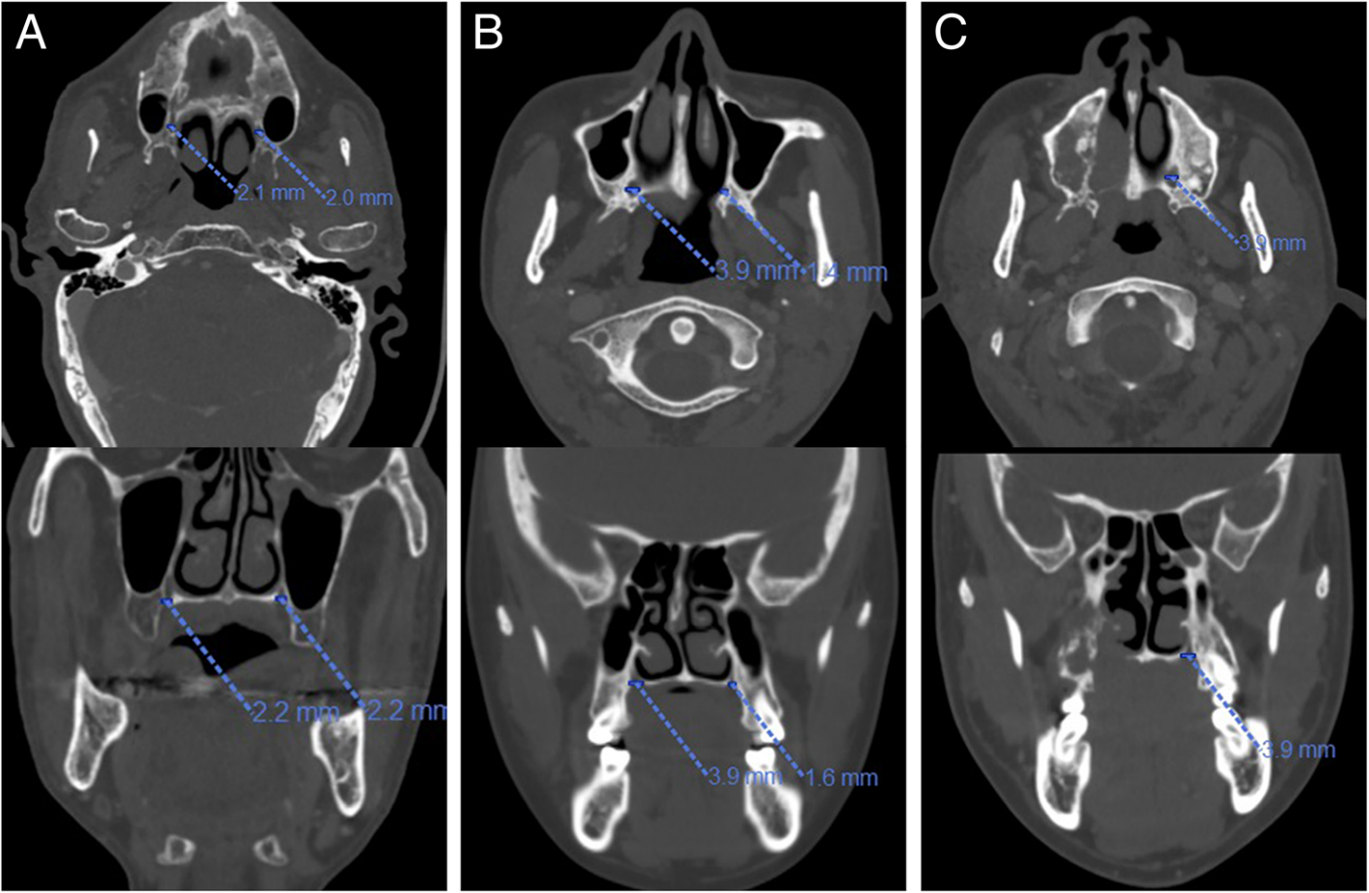
Computed tomographic images of cases with or without GPF enlargement. The diameters of the left and right GPFs were measured in bone window (upper layer: horizontal level; lower layer: coronal reconstruction). (**a**) No.35 patient was considered without GPF enlargement (GPF diameter on the tumor side was 0.2 mm larger than that on the normal side). (**b**) No.13 patient was considered with GPF enlargement (GPF diameter on the tumor side was more than 0.3 mm larger than that on the normal side). (**c**) NO. 71 patient was considered as GPF enlargement (complete destruction of GPF on the tumor side). GPF = greater palatine foramen

CT features included the tumor diameter, palatine or maxillary bone destruction, nasal cavity involvement, greater palatine foramen (GPF) enlargement, pterygopalatine fossa involvement, and foramen rotundum and cavernous sinus involvement (see Additional file 2). The average diameters of GPF on both sides in patients were determined by using horizontal level measurements, axial and coronal reconstruction was used when the horizontal level images were asymmetrical. As demonstrated by previous studies, there was minor variation (less than 0.2 mm) but no significant difference between the diameters of the left and right GPF [30, 31]. In this study, GPF enlargement was defined as a GPF diameter on the tumor side at least 0.3 mm larger than that on the normal side (Fig. 2). On the tumor side, compared with the normal side, any signs of abnormal density/signal intensity, contrast enhancement or widening of the nasal cavity, pterygopalatine fossa, foramen rotundum or cavernous sinus on the tumor side were defined as involvement. GPF enlargement, pterygopalatine fossa involvement and foramen rotundum involvement were defined as PNS signs at the same time. These criteria were consistent with those from previous studies [29, 32].

### Statistical analysis

Univariate analysis was performed in Statistical Package for the Social Sciences (SPSS) 17.0 for Windows (IBM, Armonk, NY, USA). The distributions of clinical and CT features were compared between patients with and without a diagnosis of ACC, by using the χ^2^ test (or Fisher’s exact test, when appropriate) for categorical variables and the Mann-Whitney U test for non-normally distributed continuous variables. Subsequently, a multivariable logistic regression model was established in STATA 15 for Windows (StataCorp, College of Station, TX, USA) and nomograms were constructed with the rms package in R version 3.5.0 (R Core Team, 2017). The model discrimination was evaluated by calculating the area under the receiver operating characteristic curve (AUROC). The model calibration was assessed with the Hosmer-Lemeshow goodness-of-fit test and calibration plots. Bootstrapping was used to evaluate the internal validity of the model performance measures. All statistical tests were two-sided, and P<0.05 was considered to be statistically significant.

## Results

### Pathological diagnoses of palatal tumors after surgery

Out of 102 patients, 28 patients (27%) were diagnosed with ACC by pathological examination of the surgical specimen, 40 patients (39%) were diagnosed with PA, 28 patients (27%) were diagnosed with MEC, two patients (2%) were diagnosed with LEC, and the other four patients (4%) were diagnosed with secretory carcinoma, myoepithelioma, angioleiomyoma or cystadenoma (Table 1, see Additional file 3).

**Table 1:** Number of patients with different pathological types

	Number of patients
Adenoid cystic carcinoma	28
Pleomorphic adenoma	40
Mucoepidermoid carcinoma	27
Lymphoepithelial carcinoma	2
Secretory carcinoma	1
Myoepithelioma	1
Angioleiomyoma	1
Cystadenoma	1
Total	102

### Univariate analysis of CT features and clinical symptoms

For the patients with a pathological diagnosis of ACC compared with non-ACC patients, a univariate analysis of the clinical symptoms and CT features showed that ACC patients were more likely to be older (56.21 ± 16.73 compared with 47.61 ± 16.18, *P* = 0.019); to have palatine bone destruction (27/28 compared with 33/74, P<0.001) and GPF enlargement (27/28 compared with 6/74, P<0.001); to have involvement of the pterygopalatine fossa (16/28 compared with 1/74, P<0.001), foramen rotundum (10/28 compared with 1/74, P<0.001), nasal cavity (20/28 compared with 8/74, P<0.001) and maxillary bone (18/28 compared with 9/74, P<0.001); and to have numbness (5/28 compared with 1/74, *P* = 0.007) and pain (14/28 compared with 6/74, P<0.001). There was no significant difference between ACC and non-ACC patients with regard to sex (male/female: 14/14 compared with 24/50, *P* = 0.101), tumor diameter (26.86 ± 12.33 compared with 21.15 ± 13.72, *P* = 0.057) and cavernous sinus involvement (1/27 compared with 0/74, *P* = 0.275) (Table 2).

**Table 2:** Univariate analysis of patients’ clinical information and imaging results

Variable	ACC	Non-ACC	*P* value of univariate analysis
Age (years)	56.21 ± 16.73	47.61 ± 16.18	0.019
Sex			
Male	14	24	0.101
Female	14	50	
Tumor diameter (mm)	26.86 ± 12.33	21.15 ± 13.72	0.057
Palatine bone destruction			
Yes	27	33	<0.001
No	1	41	
GPF enlargement			
Yes	27	6	<0.001
No	1	68	
Pterygopalatine fossa involvement			
Yes	16	1	<0.001
No	12	73	
Foramen rotundum involvement			
Yes	10	1	<0.001
No	18	73	
Cavernous sinus involvement			
Yes	1	0	0.275
No	27	74	
Nasal cavity involvement			
Yes	20	8	<0.001
No	8	66	
Maxillary bone destruction			
Yes	18	9	<0.001
No	10	65	
Numbness			
Yes	5	1	0.007
No	23	73	
Pain			
Yes	14	6	<0.001
No	14	68	

### Diagnostic prediction model of ACC in the palate compared with non-ACC patients

The parameters with a significant difference between ACC patients and non-ACC patients were used in multivariable logistic regression analysis. Only two predictors, age and GPF enlargement, were included in the diagnostic prediction model for ACC (see Additional file 4). The predicted probability of ACC was calculated by the model as follows (the GPF enlargement values were 1 when present and 0 when absent; see Additional file 5):

$$ \mathrm{Predicted}\ \mathrm{probability}=1/\left(1+{\exp}^{-\mathrm{risk}\ \mathrm{score}}\right) $$

$$ \mathrm{Risk}\ \mathrm{score}=-9.34+0.09\times \left(\mathrm{age}\right)+6.81\times \left(\mathrm{GPF}\ \mathrm{enlargement}\right) $$

### Evaluation of the diagnostic prediction model

**Fig. 3:**
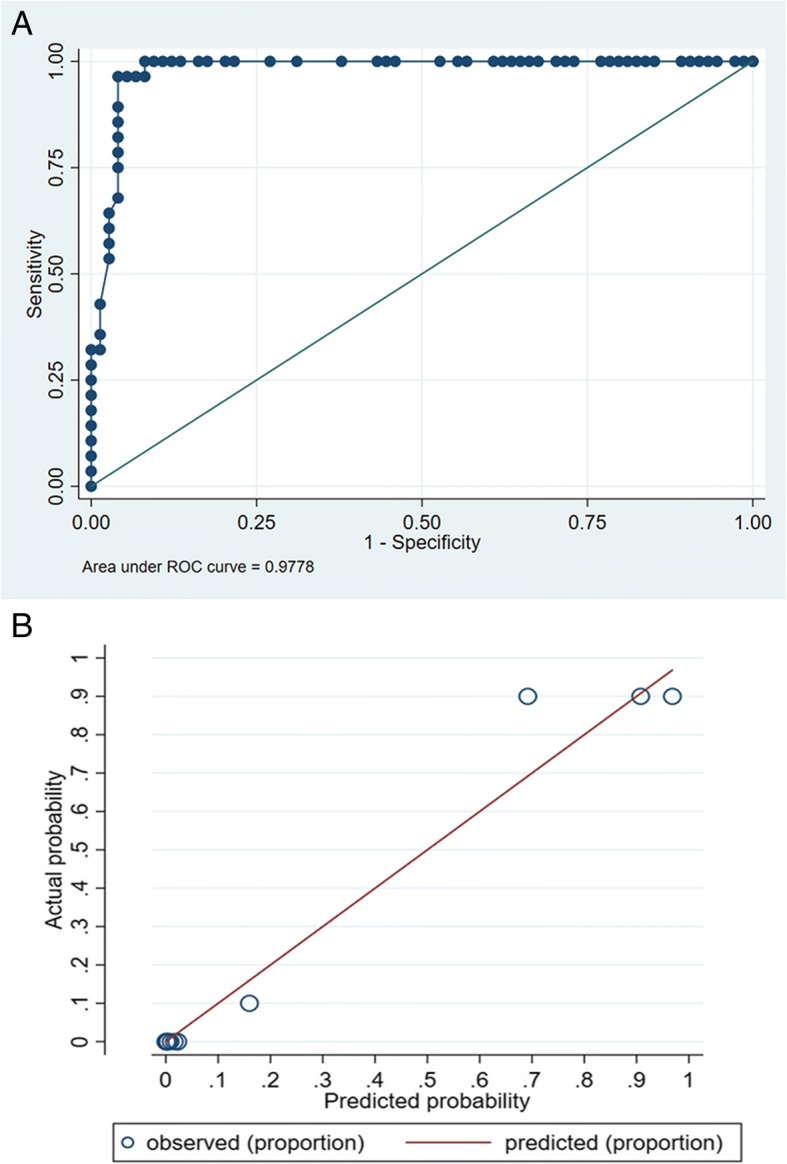
Receiver operating characteristic curve and calibration plots for the model. (**a**) The curve with dots indicated the receiver operating characteristic curve for prediction probability of adenoid cystic carcinoma. The smooth solid line indicated a non-informative area under the curve of 0.50 for comparison. (**b**) Solid line indicating perfect calibration, the model’s calibration was shown by dots

The receiver operating characteristic curve was constructed and AUROC was calculated to determine the discrimination. The Hosmer-Lemeshow goodness-of-fit test was performed to assess the calibration of the model. The model showed excellent discrimination (AUROC was 0.98; Fig. 3a) with no evidence of poor calibration (Hosmer-Lemeshow goodness-of-fit test, χ^2^ = 4.42, *P* = 0.927; Fig. 3b). One thousand random bootstrap samples with replacement from the full sample of participants were used for internal validation and the AUROC was recalculated. The AUROC based on bootstrap replicates (value = 0.98) was equal to the value from the prediction model (see Additional file 6).

### Diagnostic criteria based on age and GPF enlargement

**Fig. 4:**
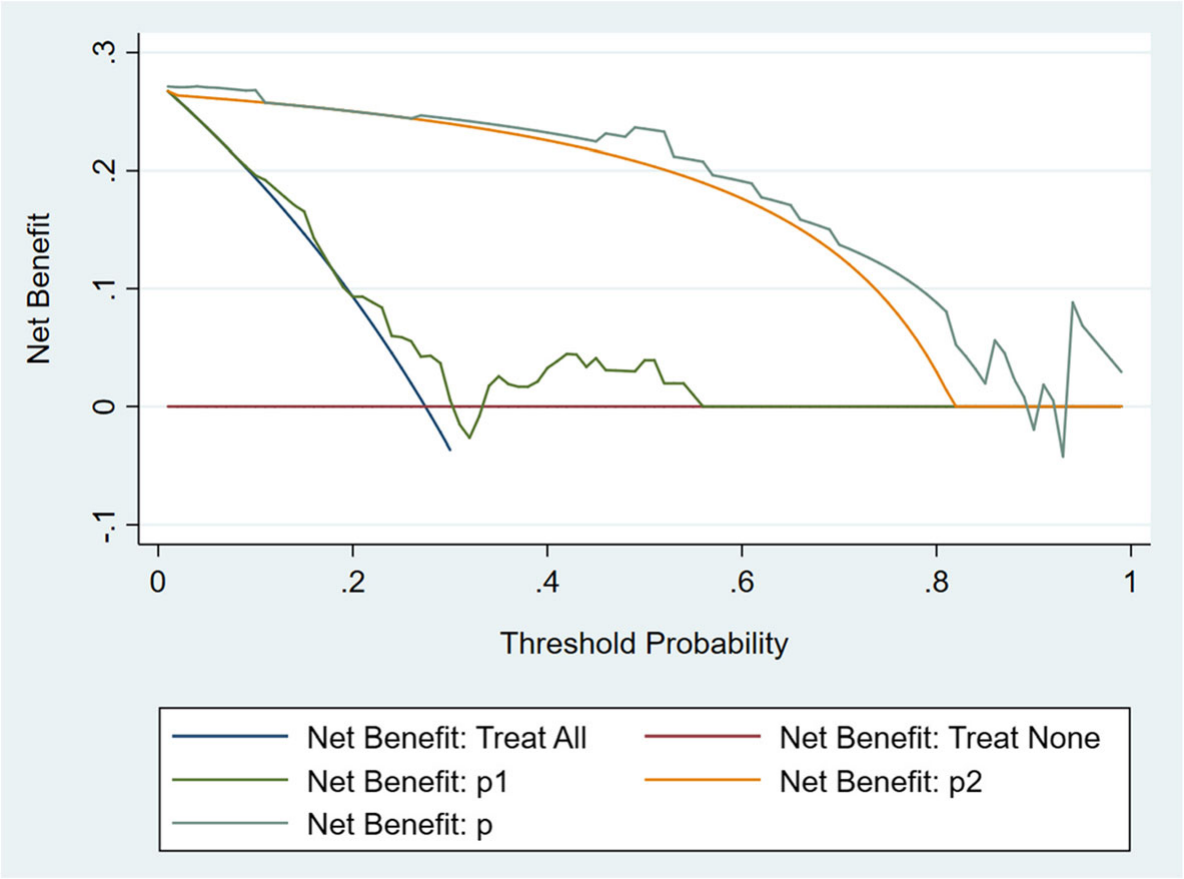
Decision curves to assign patients as positive or negative for ACC in the palate. The curve which maximized net benefit represented the optimal strategy for the associated threshold probability. The red line indicated a policy of treating no one, the blue line indicated a policy of treating all. P1 = the probability predicted by the model with factor of age alone. P2 = the probability predicted by the model with factor of greater palatine foramen enlargement alone. P = the probability predicted by the model with two factors of age and greater palatine foramen enlargement. ACC = adenoid cystic carcinoma

From the multivariable prediction model, prediction probabilities were calculated according to the sets of diagnostic criteria including the two predictors of age and GPF enlargement (Table 3), and nomograms were also constructed (see Additional file 7). The GPF enlargement variable alone had a sensitivity of 81.82% and a specificity of 98.55% (see Additional file 8), thus indicating that the presence of GPF enlargement predominated in ACC. However, the model containing two predictors, age and GPF enlargement, was considered to be the better one in a decision curve analysis, thus indicating that age should not be ignored (Fig. 4).

**Table 3:** Predicted probabilities of ACC according to age stratification with/without GPF enlargement

Predictors		Predicted probability of ACC, %	
Age (years)	GPF enlargement	GPF enlargement (+)	GPF enlargement (−)
30	+ / -	53.25	0.13
40	+ / -	73.49	0.31
50	+ / -	87.10	0.75
60	+ / -	94.27	1.79
70	+ / -	97.56	4.27
80	+ / -	98.98	9.79

## Discussion

On the basis of the CT image analysis in our study, we found that the patients with ACC in the palate with intact mucosa were more likely than the non-ACC patients to have palatine bone destruction, GPF enlargement, and involvement of the pterygopalatine fossa, foramen rotundum, nasal cavity and maxillary bone. Through multivariable logistic regression analysis, GPF enlargement was determined to be the best diagnostic predictor among the CT features of ACC in the palate with intact mucosa.

Making an accurate clinical diagnosis of the palatal mass is important in developing treatment plans. Radical resection of ACC lesions is significantly associated with survival, even after controlling for age, radiation therapy and T stage [33]. However, ACC is characterized by a high rate of perineural extension, and achieving radical resection is sometimes difficult [34]. In the palate, ACCs are generally detected at a later stage than other types of tumors, because ACCs easily invade through bone and grow into the maxillary sinus, nasal cavity, skull base or even cavernous sinus [35]. For patients with unresectable ACCs, there is a definite indication for intensity-modulated radiation therapy, and platinum-based chemoradiation regimens having a potential curative role [36]. Likewise, new insights into the biology and treatment of ACC are being widely researched [37, 38]. Obtaining an accurate pre-operative diagnosis and determining the extent of the disease are important factors in developing an appropriate treatment plan.

The predictor of GPF enlargement in this prediction model can be easily identified through CT scanning, which is suitable for most patients. GPF enlargement can also be identified through MRI [29]. Signs of PNS, including GPF enlargement and involvement of the pterygopalatine fossa and foramen rotundum, are the most characteristic features of ACC. These features have been demonstrated to affect overall survival, disease-free survival and loco-regional control of head and neck ACC patients [39]. In our univariate analysis, these PNS signs were all found to be relevant to the diagnosis of ACC, as well as some clinical features including age, numbness and pain. Univariate analysis results may provide clinicians with intuitively understood and meaningful information, but interactions can exist between these variables. Therefore, multivariable analysis was performed to obtain fewer but more precise predictive variables for the diagnosis, and GPF enlargement was found to be the best diagnostic predictor of ACC among the CT features.

Clinically, in tumors originating in the palate, GPF is the first site affected by PNS, followed by the pterygopalatine fossa and the foramen rotundum. This directivity of PNS may explain why all cases with involvement of the pterygopalatine fossa or foramen rotundum examined in this study showed an enlargement of the GPF. Therefore, GPF enlargement was the only PNS variable included in our logistic prediction model.

The variable of age was also included in our diagnostic prediction model, in agreement with previous studies indicating that ACC patients tend to be older than those with PA or MEC [11, 40, 41]. This result was confirmed by decision curve analysis; although GPF enlargement may be considered indispensable in ACC diagnosis, age should also be considered. On the basis of our prediction model, the prediction probability was greater than 87.1% in patients with GPF enlargement and an age above 50 years. Therefore, ACC should be highly suspected in older patients with GPF enlargement.

In the present study, the prediction model of clinical diagnosis for ACC in patients with palatal tumors and intact mucosa showed excellent discrimination with no evidence of poor calibration. Logistic regression analysis was used for this prediction model because it is simple, familiar to researchers, and well understood. We performed internal validation by using 1000 random bootstrap samples and obtained a favorable result. This model provides a useful clinical diagnostic tool for predicting ACC in palatal tumors with intact mucosa.

### Study limitations

There are some limitations to this study. Firstly, the sample size was not sufficiently large for us to perform external validation of the prediction model and stratification analysis of different pathological types. Secondly, because information on patients’ treatment and clinical outcomes was not recorded, the relationship between the prognosis and GPF enlargement or other CT features could not be analyzed. Therefore, external validation studies on this prediction model, with a larger sample size and more information about patients’ treatment, prognosis and clinical outcomes, should be performed in future investigations.

## Conclusions

Among the CT features, GPF enlargement is the best diagnostic predictor of ACC in the palate with intact mucosa. A prediction model of clinical diagnosis of ACC in patients with palatal tumors and intact mucosa was created and found to have satisfactory discrimination and calibration.

## Additional files

Additional file 1: Flowchart of participants in this study (TIF 9057 kb)

Additional file 2: Examples of computed tomographic features of adenoid cystic carcinoma in the palate. Computed tomographic features were identified in contrast-enhanced images. Examples of cases: the black arrow indicating: (A) palatine bone destruction, (B) nasal cavity involvement and maxillary bone destruction, (C) pterygopalatine fossa involvement, (D) foramen rotundum involvement, (E) cavernous sinus involvement. (TIF 10087 kb)

Additional file 3: Clinical summary and imaging findings of all 102 patients. (XLSX 15 kb)

Additional file 4: Result of multivariate Logistic regression analysis including all variables with significant difference from univariate analysis. (XLSX 9 kb)

Additional file 5: Result of multivariate Logistic regression analysis including age and greater palatine foramen enlargement. (XLSX 9 kb)

Additional file 6: The AUROC based on 1000 bootstrap replicates. (XLSX 9 kb)

Additional file 7: Nomogram, including age and greater palatine foramen enlargement, for patients with adenoid cystic carcinoma in the palate. The nomogram allowed to obtain the probability of adenoid cystic carcinoma in the palate according to the two predictors, age and great palatine foramen enlargement. As an example of utilization, step 1: locate the patient’s age and draw a line straight upward to the “Points” axis to determine the score associated with that age; step 2: repeat the process for the greater palatine foramen enlargement; step 3: sum the scores achieved for each covariate, and locate this sum on the “Total Points” axis; step 4: draw a line straight down to determine the likelihood of adenoid cystic carcinoma. (TIF 3444 kb)

Additional file 8: Diagnostic sensitivity and specificity of greater palatine foramen enlargement. (XLSX 9 kb)

## Abbreviations

ACC: adenoid cystic carcinoma
AUROC: area under the receiver operating characteristic curve
CT: computed tomography
GPF: greater palatine foramen
LEC: lymphoepithelial carcinoma
MEC: mucoepidermoid carcinoma
MRI: magnetic resonance imaging
PA: pleomorphic adenoma
PNS: perineural spread
SPSS: Statistical Package for the Social Sciences
