# Supplementary figures and images for: Computed tomographic features of adenoid cystic carcinoma in the palate
Source: Cancer Imaging. 2019 Jan 31;19:3. doi: 10.1186/s40644-019-0190-z (PMC6357373; doi:10.1186/s40644-019-0190-z)

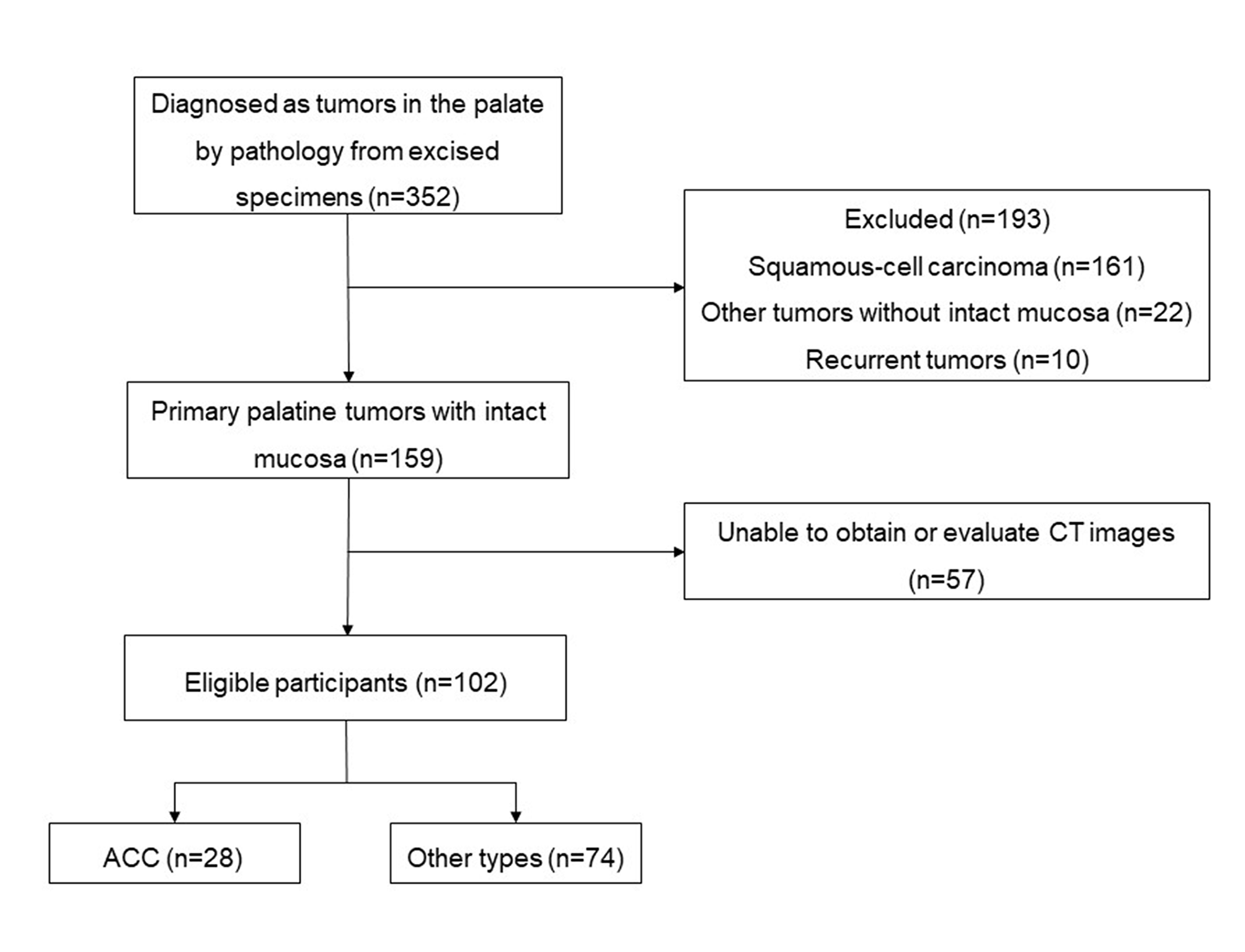

Supplement: Supplementary file 1 — Flowchart of participants in this study (TIF 9057 kb) [file 40644_2019_190_MOESM1_ESM.tif]

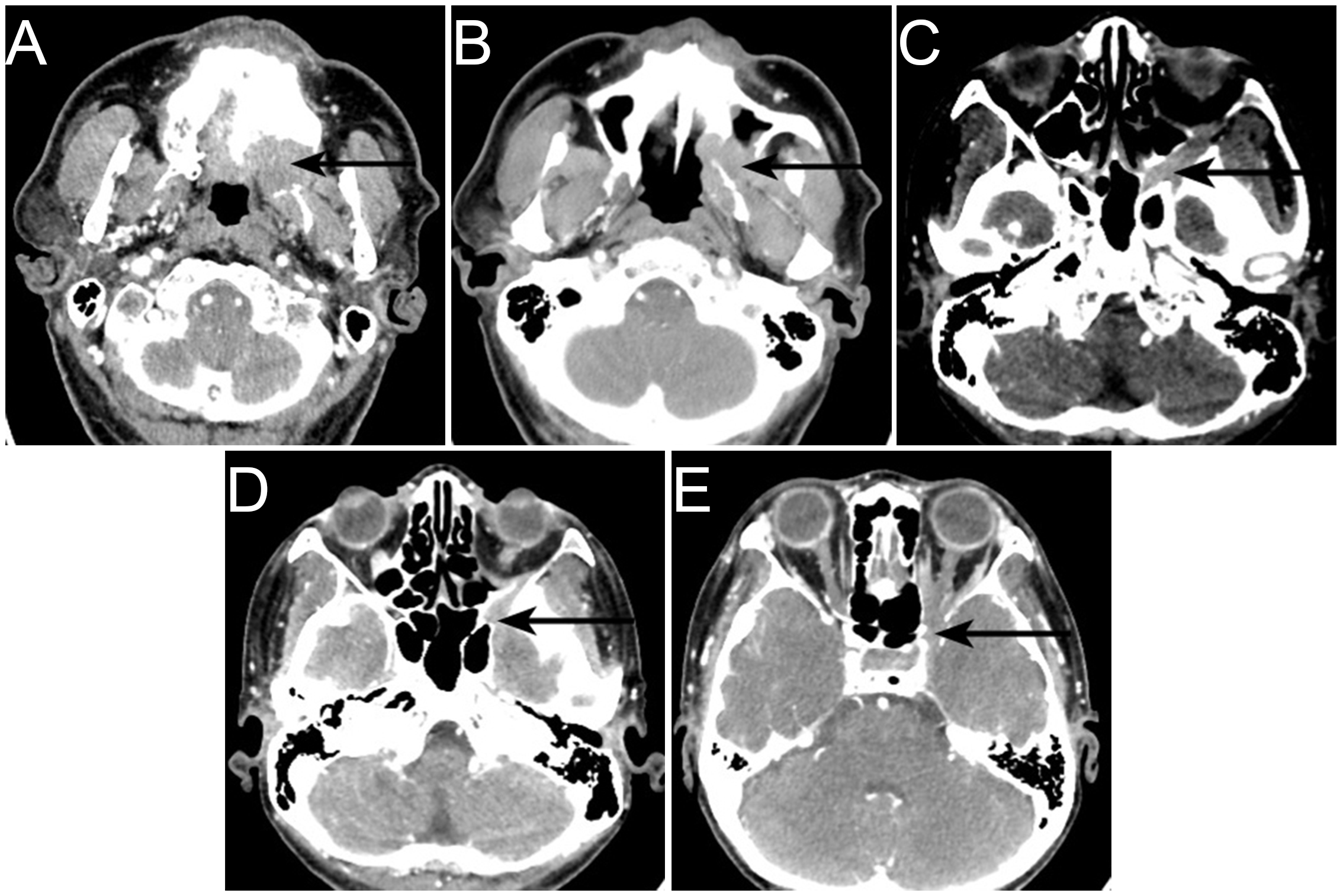

Supplement: Supplementary file 2 — Examples of computed tomographic features of adenoid cystic carcinoma in the palate. Computed tomographic features were identified in contrast-enhanced images. Examples of cases: the black arrow indicating: (A) palatine bone destruction, (B) nasal cavity involvement and maxillary bone destruction, (C) pterygopalatine fossa involvement, (D) foramen rotundum involvement, (E) cavernous sinus involvement. (TIF 10087 kb) [file 40644_2019_190_MOESM2_ESM.tif]

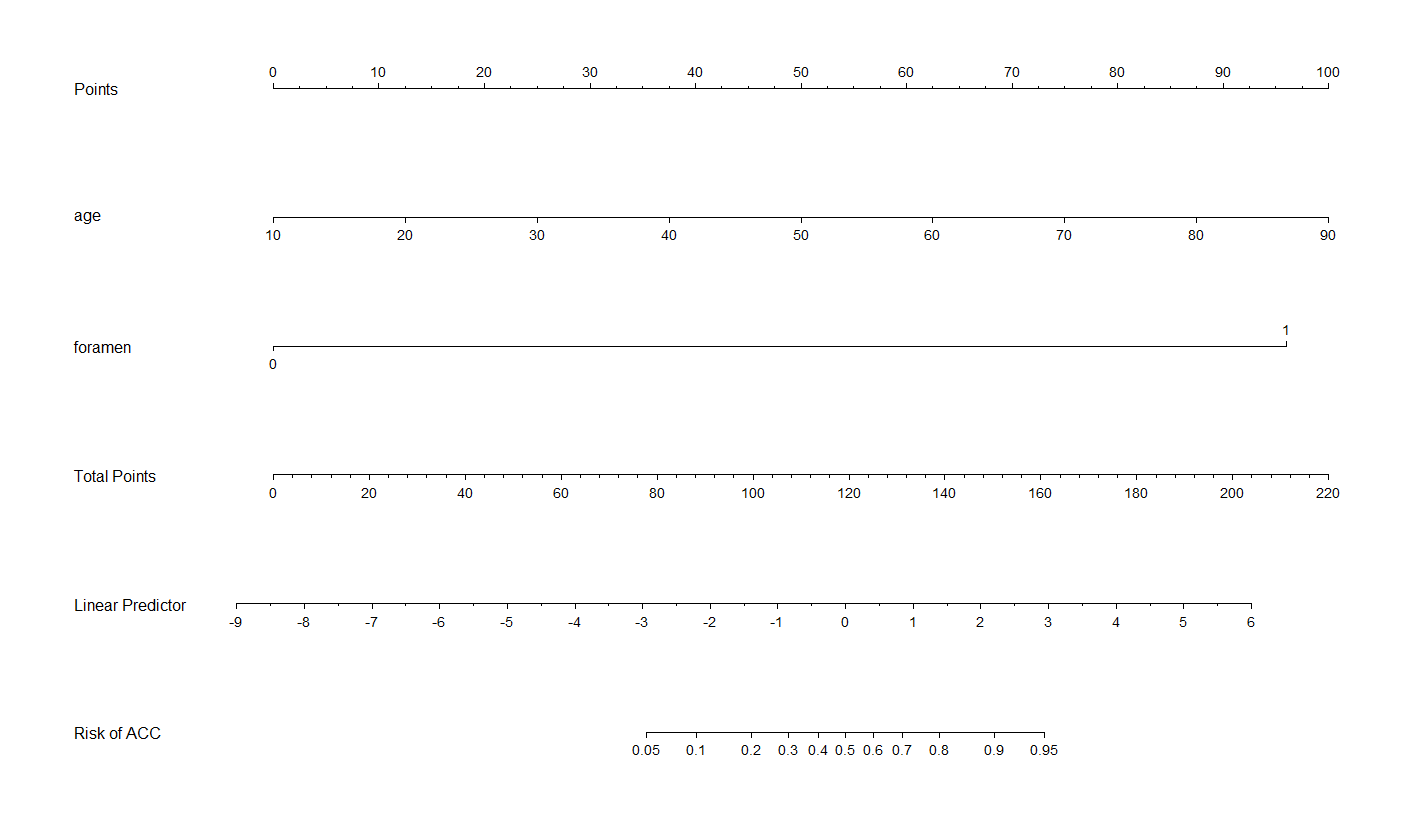

Supplement: Supplementary file 7 — Nomogram, including age and greater palatine foramen enlargement, for patients with adenoid cystic carcinoma in the palate. The nomogram allowed to obtain the probability of adenoid cystic carcinoma in the palate according to the two predictors, age and great palatine foramen enlargement. As an example of utilization, step 1: locate the patient’s age and draw a line straight upward to the “Points” axis to determine the score associated with that age; step 2: repeat the process for the greater palatine foramen enlargement; step 3: sum the scores achieved for each covariate, and locate this sum on the “Total Points” axis; step 4: draw a line straight down to determine the likelihood of adenoid cystic carcinoma. (TIF 3444 kb) [file 40644_2019_190_MOESM7_ESM.tif]
